# Elaboration and characterization of molybdenum titanium tungsto-phosphate towards the decontamination of radioactive liquid waste from ^137^ Cs and ^85^Sr

**DOI:** 10.1007/s11356-023-31104-4

**Published:** 2023-12-08

**Authors:** Ezzat A. Abdel-Galil, Abeer E. Kasem, Sara S. Mahrous

**Affiliations:** Environmental Radioactive Pollution Department, Hot Laboratories and Waste Management Centre, Egyptian Atomic Energy Authority, Cairo, Egypt

**Keywords:** Inorganic sorbents, Cesium, Strontium, Wastewater, Sorption, MoTiWPO_4_

## Abstract

The crystalline phase of molybdenum titanium tungsto-phosphate (MoTiWPO_4_) as an inorganic sorbent material was synthesized via the sol–gel method. The physicochemical characteristics of MoTiWPO_4_ were evaluated by using Fourier transform infrared (FT-IR), scanning electron microscope (SEM), energy dispersive X-ray (EDX), thermal analysis (TGA-DTA), and X-ray diffraction (XRD). MoTiWPO_4_ sorbent material exhibits a high chemical resistance to HNO_3_, HCl, and alkaline media. MoTiWPO_4_ has good thermal stability as it retained about 75.63% of its saturation capacity upon heating at 500 °C. The sorption studies for several metal ions revealed marked high sorption efficiency of MoTiWPO_4_ towards Cs^+^ and Sr^2+^ ions which reached 99% and 95%, respectively. The saturation capacity of MoTiWPO_4_ for Cs^+^ and Sr^2+^ is 113 and 109 mg/g, respectively. MoTiWPO_4_ is approved to be successfully eliminating both ^137^Cs and ^85^Sr from liquid radioactive waste streams by %eff. of 92.5 and 90.3, respectively, in the presence of competing ions from ^60^Co(divalent) and ^152^Eu (trivalent), confirming the batch experiment results for the removal of Cs^+^ and Sr^2+^ metal ions. Furthermore, the decontamination factor exceeds 13.3 in the case of ^137^Cs and 10.3 for ^85^Sr.

## Introduction

The operation of nuclear power plants, reprocessing facilities, and research centers, as well as the utilization of radioisotopes in industry and medical diagnostics, all result in the generation of various radioactive wastes. The radioisotopes of strontium and cesium are classified as the most harmful isotopes produced in the nuclear industry due to their long half-life, high chemical activity, migration, toxicity, and relatively small atomic radii (Nasseh et al. 2017; El-Din et al. 2019; Mahrous et al. 2022b).

Developing new or conducting existing treatment methodologies is considered an essential step in nuclear waste safety (Yao et al. 2022). Environmental contamination produced by the periodic discharge of effluents from industrial waste into geo and biospheres has become a global catastrophe, especially in this century. This is owing to the presence of numerous contaminants in these aqueous radioactive and industrial waste streams, which have a severe influence on the health of people and the environment (Feng et al. 2022; Stäger et al. 2023). For example, the spread of these contaminants may reach the groundwater leading to the unstoppable spread of contamination pathways through food and water chains. The contamination spread will produce harmful health effects on humans, animals, and plants. The cancer disease will spread above its normal rate; moreover, the plant and animal growth will be affected making large consequences on the environment (El-Aryan et al. 2014; Yang et al. 2021; Sopapan et al. 2023).

An applicable solution for decreasing the amount of the generated industrial and radioactive waste and treatment methodologies shall take the attention of researchers in the field of radioactive waste management. In the past decades, numerous methodologies have been developed to treat industrial and radioactive liquid waste.

One of the most common treatment processes is solvent extraction used by Patra et al., (2022) for individual extraction of ^137^Cs and ^90^Sr via 1,3-di-octyloxycalix arene-crown-6 (CC6) and dicyclohexano-18-crown-6 [DCH18CH]. Lee et al., (2022) used a flocculation method based on an inorganic washing solution to remove Cs^+^ and Sr^2+^ from the soil, while co-precipitation was used by Sopapan et al., (2023) via the application of potassium ferrocyanide to remove ^134,137^Cs from low and intermediate level liquid radioactive waste. Also, another advanced technology such as membrane separation, ion exchange, and adsorption has been carried out for the removal of cesium and strontium radionuclides (El-Nagaar et al. 2012; El-Aryan et al. 2014; Rizk and Hamed 2015; Li et al. 2017; Nasseh et al. 2017; Abdel-Galil et al. 2018, 2020, 2021; Mahrous et al. 2019, 2022a, c; Amesh et al. 2020; Pham et al. 2020; Akinhanmi et al. 2020; Bediako et al. 2020; Cheng et al. 2020; Yang et al. 2021; Abass et al. 2022; Lee et al. 2022; Patra et al. 2022; Yao et al. 2022; Șenilă et al. 2023; Sopapan et al. 2023) using organic, inorganic, or composite materials.

Adsorption is widely considered to be the most efficient and practical approach for eliminating metal ions from aqueous solutions. This is due to the improvement of economical adsorbents and adsorbent precursors, as well as their simplicity of handling and operation (Yang et al. 2021). To eliminate metal ions from radioactive and non-radioactive wastewater, several organic and inorganic sorbent materials have been studied. Inorganic materials that are naturally occurring or created synthetically have long been used as adsorbents for different radionuclides, primarily alkali and alkaline earth cations (Abdel-Galil et al. 2018; Bediako et al. 2020; Hai et al. 2020). Due to their strong radiation resistance, thermal and mechanical stability, and compatibility with ultimate waste forms, it is suitable for radioactive waste disposal (Pandiarajan et al. 2018; El-Din et al. 2019; Akinhanmi et al. 2020). Several synthesized inorganic sorbents have significant affinities for one or more radionuclides throughout a broad pH range. These sorbents are adaptable and may be utilized in two different ways, either in granular form in a typical packed-bed installation or in a finely split form in combination with an effective solid–liquid separation method like cross-flow filtering (El-Aryan et al. 2014).

In this study, the preparation of molybdenum titanium tungsto-phosphate sorbent material has been carried out for the selective removal for ^137^Cs and ^85^Sr from liquid radioactive waste streams. The prepared sorbent material is based on the use of inorganic metals such as molybdenum, titanium, tungsten, and phosphate. The saturation capacity, radiation, and thermal stability for MoTiWPO_4_ sorbent material were investigated. Thermodynamic parameters (Δ*G*^0^, Δ*S*^0^, and Δ*H*^0^) for the adsorption of Cs^+^, Sr^2+^ onto molybdenum titanium tungsto-phosphate were also calculated. Moreover, the application of MoTiWPO_4_ in the sorption of ^137^Cs and ^85^Sr from simulated liquid radioactive waste sample in the presence of ^60^Co and ^152^Eu as competing ions was also studied.

## Experimental

Orthophosphoric acid, sodium tungstate, titanium tetrachloride, and ammonium molybdate are made by Sigma-Aldrich and utilized without additional purification.

### Radiotracers

Radiotracers of ^85^Sr,^60^Co and ^152+154^ Eu were obtained via the activation reaction (*n*, *γ*) by the neutron irradiation of 0.05 g weight of metal chloride salt double wrapped in aluminum foil and transferred via the rabbit system to the vertical irradiation channels inside the core of the second Egyptian Training Research Reactor ETRR-2. ^137^Cs was obtained from a standard radioactive solution purchased from Eckert and Ziegler.

The radioactivity level of the activated samples was measured using coaxial-p type HPGe detector (Canberra, USA) connected to multi-channel analyzer with 16,000 channels (Canberra, USA). The obtained results were analyzed using Gennie 2000 software after energy and efficiency calibration.

### Preparation of chemical reagents

In double-distilled water (DDW), orthophosphoric acid (1.0 M) H_3_PO_4_ and sodium tungstate (0.1 M) Na_2_WO_4_·2H_2_O were prepared, while (0.1 M) titanium tetrachloride (TiCl_4_) was formed in 4.0 M HCl. A solution of 0.1 M ammonium molybdate ((NH_4_)_6_Mo_7_O_24_) (dissolved in deionized water) was prepared; also, 0.1 M NH_4_OH to adjust the pH of the solution was diluted.

### Preparation of molybdenum titanium tungsto phosphate (MoTiWPO_4_)

At 25 ± 1 °C, an inorganic precipitate of molybdenum titanium tungsto phosphate was prepared by mixing 250 mL orthophosphoric (1 M) and 250 mL sodium tungstate (0.1 M) solutions with stirring for 30 min to create a homogeneous pale-yellow solution. Then, 250-mL ammonium molybdate (0.1 M) was added to the mixture with stirring for another 30 min. The obtained mixture was then added to 250-mL titanium tetra chloride (0.1 M) with stirring for at least 1 h. Aqueous ammonia was used to alter the pH to 0.9 and stirring until a green precipitate appeared. The resulting green slurry was left to digest for 24 h at room temperature. After centrifuging the gel, and decanting the supernatant liquid, it was dried at 50 ± 1 °C. The produced precipitate was crushed to get small granules before being converted to H^+^ form by treating with 1.0 M HNO_3_ for 24 h with intermittent shaking and replacing the supernatant liquid with new acid. After multiple washes with DDW, the excess acid was removed; the particles were dried at 50 ± 1 °C and sieved to achieve particles of a specific size range (0.12–0.75 mm).

### Characterization of MoTiWPO_4_

The prepared MoTiWPO_4_ was examined via several characterization techniques. To examine the surface morphology, scanning electron microscopy, or SEM (JEOL-JSM-6510LA, Tokyo, Japan), was employed. With the use of a Cu-k target and an X-ray diffraction system (Shimadzu XD-D1, Japan), crystal structure, crystalline size, and phase were examined. Using an Oxford Inca EDX detector for compositional analysis, the energy-dispersive X-ray analysis EDX model (JSM-5600 LV, JEOL, Japan) was used for the elemental analysis. A thermogravimetric analysis TGA-DTA test (Shimadzu DTG-60/60H-Thermal Analyzer, Japan) was used to assess the thermal stability. Brunauer, Emmett, and Teller (BET) measurements (Quanta chrome Novawin-Data Acquisition and Reduction for NOVA instruments) were used to obtain the specific surface area, pore volume, and pore size of MoTiWPO_4._ Last but not least, the active function groups were discovered utilizing FT-IR analysis (FT-IR, Nicolet spectrometer, Meslo, USA.

### Adsorption experiments

Batch tests were performed to determine if the MoTiWPO_4_ could effectively adsorb Cs^+^ and Sr^2+^ species from wastewater. The appropriate cation species solution and 50 mg of powdered MoTiWPO_4_ were used in 15-mL glass bottles for the experiments. For the investigations, a thermostatic shaker with 250 rpm was used to shake the glass containers after they had been sealed; the ambient temperature was set at 298 K. To change the pH, the proper volume of 0.1 M HCl or NH_4_OH was applied. Water that has been twice distilled was utilized to make the experiment’s solutions. Experiments using solutions with concentrations between 50 and 500 mg L^−1^ to investigate the impact of initial adsorbate concentration were utilized. A contact time of 10 to 300 min is also included. The following formula was used to obtain the removal effectiveness (%R):

1 $$\%R=\frac{({C}_{o}-{C}_{f})}{{C}_{o}}\times 100$$

The adsorption capacity at any time (*q*_*t*_) and at equilibrium (*q*_*e*_) may be calculated using the following formula:

2 $${q}_{e}=({C}_{o}-{C}_{f)}\times \frac{V}{m} \, (\text{mg/g})$$

and

3 $${q}_{t}=\left({C}_{o}-{C}_{t}\right)\times \frac{V}{m} \, (\text{mg/g})$$

The decontamination factor (DF) is determined as follows:

4 $$DF=\frac{{A}_{o}}{{A}_{f}}$$

### Saturation capacity and thermal effect

Heating the MoTiWPO_4_ at different drying temperatures (100, 300, 500, and 700 °C) for 4 h in a muffle furnace was used to study the effect of heating on the saturation capacity of the produced sorbent material. Following that, 50 mg of previously heated MoTiWPO_4_ was placed in a glass container with 5 mL Cs^+^ ion solution (300 mg/L) at *V*/*m* = 100 mL/g. For 24 h, the mixture was constantly shaken at 250 rpm in a temperature-controlled incubator shaker (Kottermann D-1362, Germany) until equilibration was achieved. The solution was filtered after achieving equilibrium, and the concentration of Cs^+^ ions was determined using an atomic absorption spectrophotometer. This process has been done several times with a new Cs^+^ ion solution or until complete saturation of the prepared MoTiWPO_4_ with Cs^+^ ions. The saturation capacity value of MoTiWPO_4_ (mg/g) was calculated by using the following equation:

5 $$\text{Capacity }(\text{mg/g})=({C}_{o}-{C}_{f})\times\frac{V}{m}$$

where *C*_*o*_ and *C*_*f*_ represent the concentrations of the ions in solution before and after equilibrium (mg/L), respectively, *V* is solution volume (L), and *m* is the mass of the sorbent material(g).

## Results and discussion

### Characterization

#### SEM and EDX

**Fig. 1:**
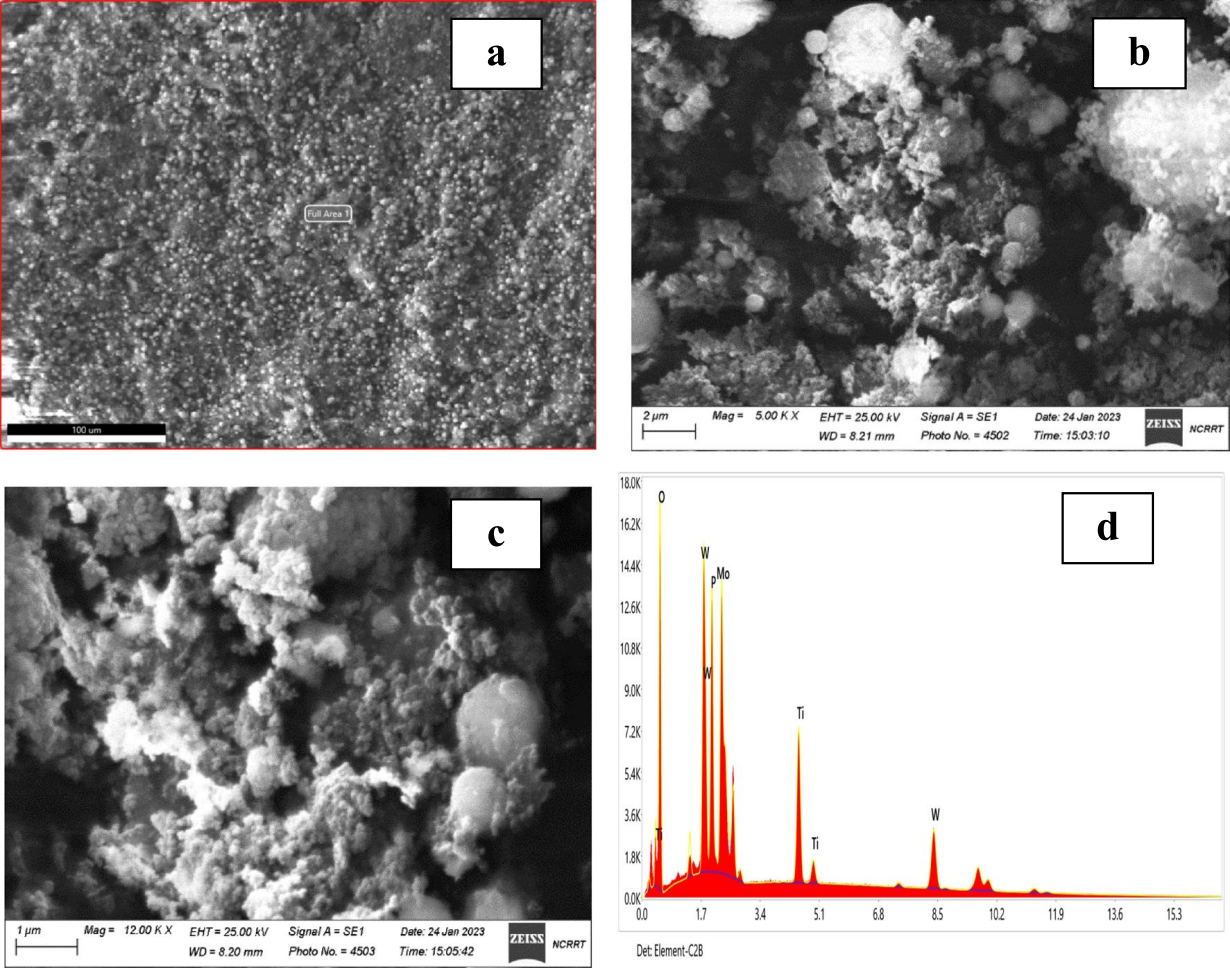
SEM and EDX for MoTiWPO_4_: **a**) the full surface at 100-µm particle size and low magnification power, **b**) × 5000 and **c**) × 12,000; **d**) is the x-ray dispersive energy spectrum from EDX analysis

As described in Fig. 1 a, b, and c, the SEM micrographs along with EDX spectrum (d) for the prepared MoTiWPO_4_ sorbent material. The surface morphology that appears in Fig. 1a demonstrates the microstructure and homogeneity of particle distribution at low magnification powers.

**Fig. 2:**
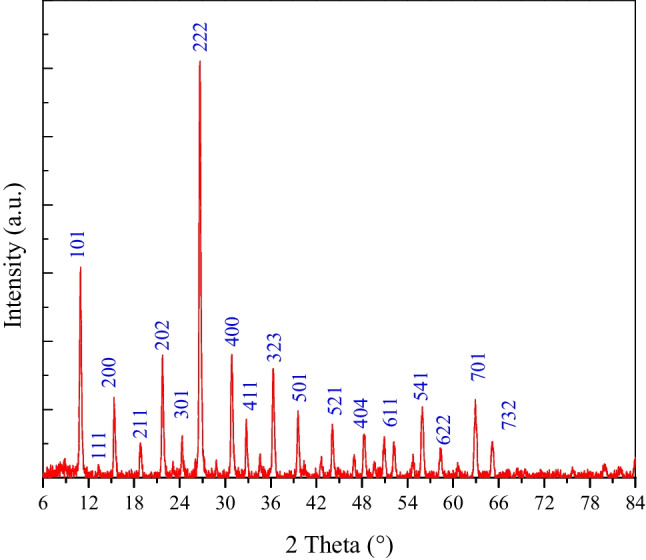
XRD pattern for MoTiWoPO_4_

Figure 2 b and c illustrate the multi-component cavities, gaps, and cracks are created at the inertial distances between the components of the MoTiWoPO_4_, resulting in the overall high surface heterogeneity, which may be a factor to increase the efficiency of the sorbent material to decontaminate high percent of ^137^Cs and ^85^Sr from liquid radioactive waste. The percentages of the elements for MoTiWPO_4_ are revealed in Fig. 1d, obtained by the EDX method, and are stated quantitatively in Table 1.

**Table 1:** Elemental composition of MoTiWPO_4_ sorbent material

Element	Molybdenum titanium tungsto phosphate	
	Element (%)	Atomic (%)
O	18.97	68.89
p	11.13	8.28
Ti	8.3	4,41
Mo	30.3	5.68
W	22.9	2.75

#### XRD

Figure 2 shows that MoTiWPO_4_ has a crystalline nature, which is expected for inorganic materials. Also, the pattern shows many intense peaks at different 2θ value ranges of (10–65°) (Akinhanmi et al. 2020). Using Match (Phase analysis using powder diffraction X-ray) version 3.14 (Germany) software, analysis and matching of the obtained experimental data are conducted. The result of the analysis suggests that the most probable matching occurs with diffraction card no. 96–221-0408 from (crystallography open database) COD, given the crystal structure of a cubic with space group Pn-3m (224). The calculated lattice parameter is *a*, *b*, *c* = 11.66078 Å and *α*, *β*, *γ* = 90°. In addition, the crystalline dimension (*D*, nm) for MoTiWPO_4_ was calculated using the Bragg angle (*θ*) and the full width at half maximum (FWHM) by using Debye-Scherer’s equation:

6 $$D=\;\frac{K\;\lambda }{\beta\;Cos\theta }$$

where *K* and *λ* are the shape factor (0.9) and the wavelength of Cu–K_α_ radiation (*λ* = 0.15406 nm), respectively. The calculated average crystallite size is 51.62 nm. The degree of crystallinity of the prepared MoTiWPO_4_ is 97.94%.

#### FT-IR

**Fig. 3:**
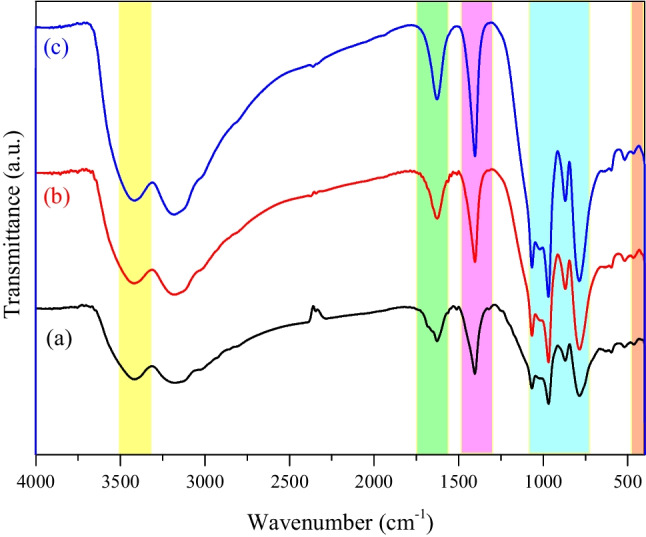
FT-IR spectra for MoTiWPO_4_ sorbent material as a function of wavenumber (cm^−1^) ((a) MoTiWPO_4_, (b) MoTiWPO_4_ loaded with Cs^+^, and (c) MoTiWPO_4_ loaded with Sr.^2+^)

The FT-IR of MoTiWPO_4_ transmission spectra appears in Fig. 3 and Table 2. Figure 3 (a) shows significant band absorption is about 3420 cm^−1^ equivalent to the stretching vibration of O–H groups of external adsorbed water (Mahrous et al. 2019). The peak observed at the peak at ≈ 1626cm^–1^ indicates the water molecules’ bending mode (El-Aryan et al. 2014). The peak at 1403cm^–1^ corresponds to the metal–OH bond. The phosphate group appears at 785–1066 cm^−1^ (El-Aryan et al. 2014). The IR spectrum of MoTiWPO_4_ loaded with Cs^+^ and Sr ^2+^ is also given in Fig. 3(b and c) and Table 2. The new peak appears at 453–468 cm^−1^ which can be assigned to metal–oxygen bonds (Mahrous et al. 2022c). From Fig. 3(b and c), a change has been observed in the intensity and wavenumbers of the O–H and phosphate group which may be given to indicate that these functional groups are involved in the sorption process.

**Table 2:** FT-IR wavenumbers of MoTiWPO_4_, MoTiWPO_4_/Cs^+^, and MoTiWPO_4_/Sr^2+^

Function group	MoTiWPO_4_	MoTiWPO_4_/Cs^+^	MoTiWPO_4_/Sr^2+^
ν(OH)	3420	3417	3417
δ(H2O)	1626	1627	1628
PO4^3−^	785–1066	786–1066	787–1066
M–O	––––	453	468

#### Thermal analysis TGA-DTA

**Fig.4:**
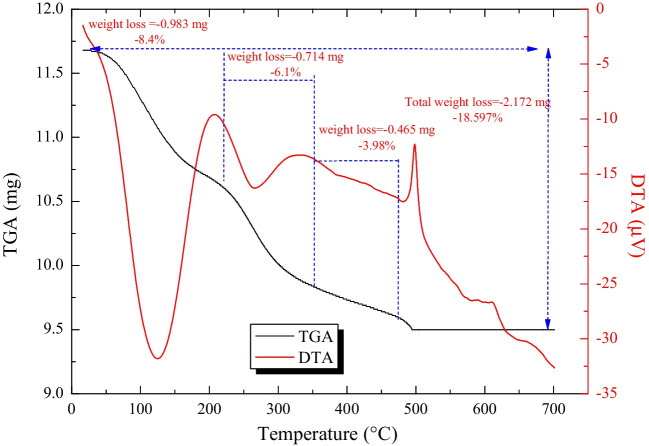
TGA-DTA for the MoTiWPO_4_ sorbent material

The TGA-DTA profile of MoTiWPO_4_ is shown in Fig. 4. According to the TGA investigation of MoTiWPO_4_, there are three different areas where mass is lost up to 700 °C. The initial mass loss owing to humidity is reflected in the first area through heating to 200 °C (Abdel-Galil et al. 2021) where the weight loss is 8.4%. Its corresponding endothermic peak in the DTA curve is observed at 125.07 °C. The second mass loss region observed a weight loss of 6.11% between 200 and 350 °C which may be associated with the condensation of the phosphate group to pyrophosphate groups. Its corresponding endothermic peak in the DTA curve is observed at 250 °C (El-Aryan et al. 2014).

Further weight loss of 3.98% on heating to 490 °C may be due to the elimination of interstitial water molecules from the material (Abdel-Galil et al. 2021). The exothermic peak in the DTA at 500 °C may be due to the structural transformation of the material without weight loss (El-Nagaar et al. 2012)**.**

#### Surface area

Surface area is one of the most important characteristics of adsorbents, as the higher its value, the greater the ability of the adsorbent materials to adsorb metal ions from wastewater (Li et al. 2017). For MoTiWPO_4_, it exhibits an S_BET_ value of ~ 39.5 m^2^/g, a total pore volume of ~ 3.2 × 10^−2^ cc/g, and an average pore size of ~ 1.6 Å. For comparison with other inorganic sorbent materials, Table 3 lists the compared inorganic materials along with the specific surface area computed by the BET method.

**Table 3:** Comparison between the BET calculation of surface area for different inorganic sorbent materials

Reference	Material	Surface area (m^2^ g^−1^)	Pore volume (cm^3^ g^−1^)	Pore diameter (nm)
Hassan et al. (2019)	Mg/Fe Hydrotalcite	92.1	0.21	11.0
Mansy et al. (2017)	AlSiO_2_/Mg	0.6	4.5E-04	14.2
Vasiliev et al. (2019)	Termoxide-39	17.0	––	––
Solińska and Bajda (2022)	Clinoptilolite	28.2	––	––
Solińska and Bajda (2022)	Clinoptilolite / HDTMA-Br	14.5	––	––
Present work	MoTiWPO_4_	39.5	3.2E − 02	16.3

Table 3 shows that the present work sorbent material has an intermediate surface area value between the other compared inorganic sorbents. These results enhance its application for the removal of the studied metal ions.

#### Point zero charge (PZC)

**Fig. 5:**
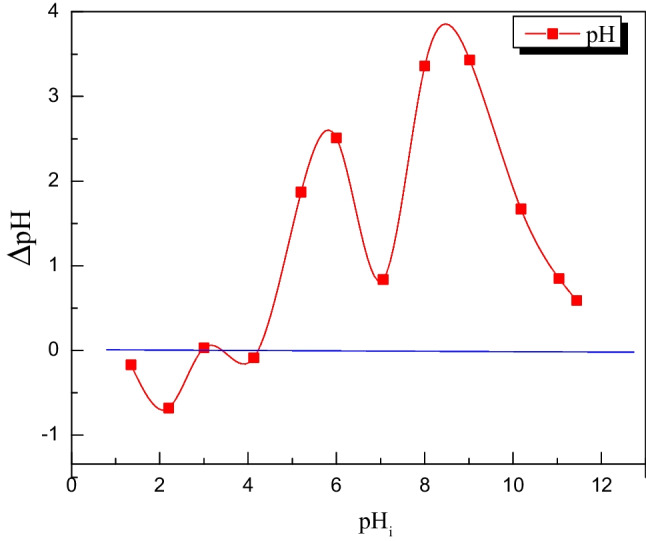
Point zero charge for MoTiWPO_4_

Point zero charge is the pH value at which the sorbent material’s surface has no charges (Abass et al. 2022). The surface of MoTiWPO_4_ has a positive charge below this pH level to interact with negative species. The negatively charged surface above it interacts with positive species. The pH_PZC_ for MoTiWPO_4_ is 2.9 as shown in Fig. 5; this value is compatible with the sorption data as will be seen later.

#### Chemical dissolution

According to Table 4, the synthesized MoTiWPO_4_ sorbent material exhibits a high chemical stability in DDW, acids (HNO_3_ and HCl), and bases (NaOH and KOH). This finding demonstrated that the MoTiWPO_4_ may be employed in both acidic and basic media, particularly at low concentrations.

**Table 4:** The dissolution of MoTiWPO_4_ in various solvents at 25 ± 1 °C

Sorbent material	Media	Concentration (M)	Solubility (g/L)
MoTiWPO_4_	H_2_O	–-	B.D
	HNO_3_	0.1	0.17
		0.5	0.19
		1.0	0.20
		3.0	0.24
		5.0	0.35
	HCl	0.1	0.19
		0.5	0.22
		1.0	0.24
		3.0	0.28
		5.0	0.31
	NaOH	0.1	0.28
		1.0	0.44
	KOH	0.1	0.35
		1.0	0.43

### Adsorption batch experiments

#### Effect of the adsorbent amount

**Fig. 6:**
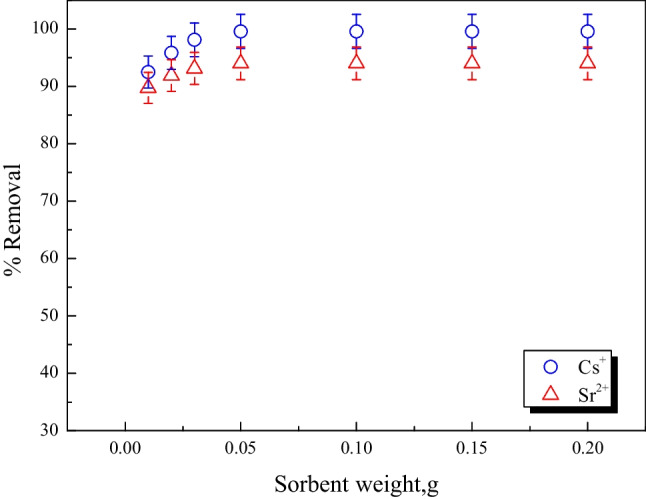
The effect of adsorbent amount on Cs^+^ and Sr^2+^ removal by MoTiWPO_4_

The impact of adsorbent quantity on the removal of Cs^+^ and Sr^2+^ was investigated, and the results are given in Fig. 6. The removal of Cs^+^ and Sr^2+^ behaves similarly, rising with an increase in adsorbent quantity until equilibrium is achieved at the adsorbent quantity greater than 0.05 g as shown in Fig. 6. This pattern can be explained by the fact that when sorbent amounts rise, more active sites become available to adsorb more metal ions until equilibrium is achieved (El-Nagaar et al. 2012; Mansy et al. 2017; El-Din et al. 2019; Hassan et al. 2019; Abdel-Galil et al. 2020; Abass et al. 2022; Mahrous et al. 2022a). As a result, the optimal adsorbent dose for attaining the maximum percent removal is 0.05 g/5 mL.

#### Effect of initial concentration

**Fig. 7:**
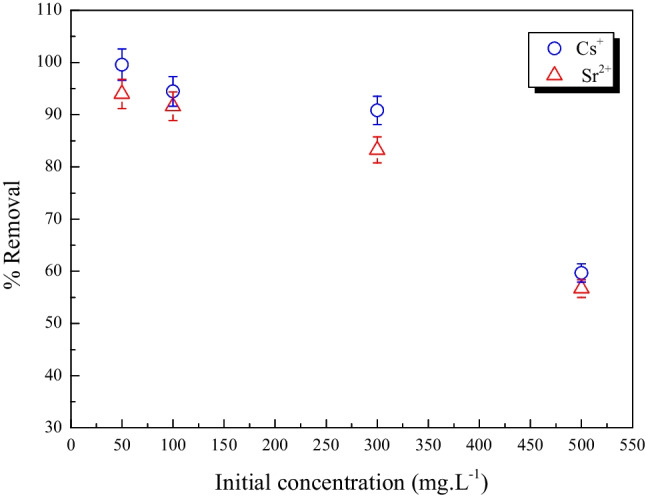
The effect of concentration of Cs^+^ and Sr^2+^ removal by MoTiWPO_4_

The influence of the adsorbate’s initial concentration was investigated by conducting the adsorption experiment with various beginning concentrations of Cs ^+^ and Sr^2+^ (50–500 mg L^−1^) until equilibrium was established; the findings are shown in Fig. 7.

The results reveal that a high percentage of removal was observed at lower concentrations of Cs^+^ and Sr^2+^ while at high concentrations of cations, the % removal decreased. This result is mostly attributable to the effective adsorbent surface’s adsorption site saturation, in which there are accessible unoccupied sites at low concentrations. As the primary Cs^+^ and Sr^2+^ concentration was raised, the essential active sites for the cations adsorption were rapidly occupied, so the % removal decreased as the concentration increased (Rizk and Hamed 2015)**.**

#### Effect of pH

**Fig. 8:**
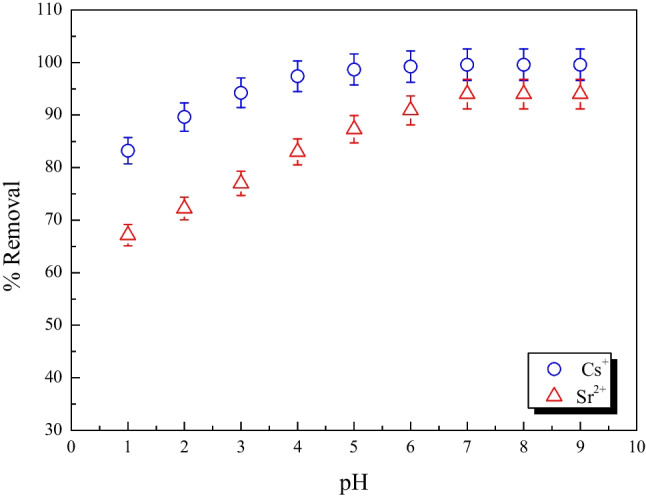
The effect of solution pH on removing Cs^+^ and Sr^2+^ by MoTiWPO_4_

The most significant variable affecting metal ions adsorption onto the adsorbent surface has been identified as aqueous solution pH. This is due to a portion of hydrogen ions themselves competing with adsorbates (Pham et al. 2020). Figure 8 depicts the influence of pH on the removal of Cs^+^ and Sr^2+^ ions from aqueous solutions by MoTiWPO_4_. It is evident that the Cs^+^ and Sr^2+^ adsorption efficiency improved quickly with the pH change from 1 to 6. Following that, the rise in adsorption efficiency is gradual until it reaches equilibrium at a pH of 7. So, in order to get the highest adsorption efficiency, a pH of 7 was chosen as the ideal.

#### Effect of contact time

**Fig. 9:**
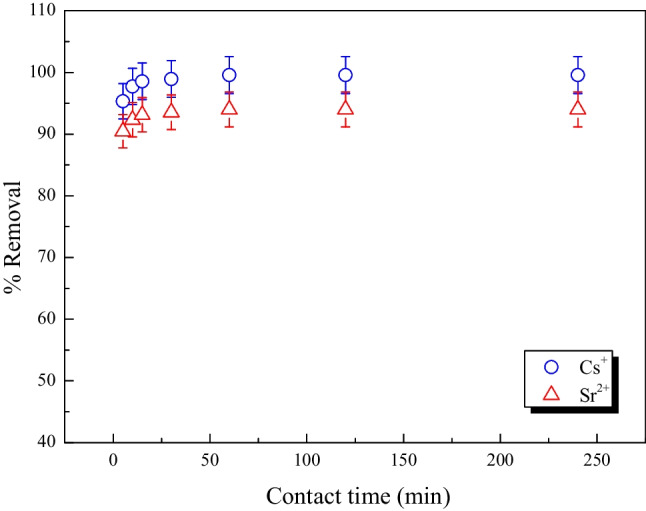
The effect of contact time in removing Cs^+^ and Sr^2+^ by MoTiWPO_4_

Contact time is a major factor in all transfer phenomena, including adsorption. Therefore, it is essential to study how it affects the % removal of Cs^+^ and Sr^2+^. By carrying out an adsorption test with a starting concentration of 50 mg L^−1^ at various contact durations ranging from 10 to 300 min, the impact of contact time on the adsorption process was investigated. The data shown in Fig. 9 demonstrate that the efficiency of Cs^+^ and Sr^2+^ adsorption onto the MoTiWPO_4_ increased with increasing contact time.

The beginning region of the curve showed fast adsorption, which corresponding to the adsorption of the Cs^+^ and Sr^2+^ on the sites that could be reached quickly on the outside edges of the surface of the sorbent material (Nasseh et al. 2017). When the contact time approached equilibrium, a little decline in adsorption was shown in the second portion of the curve. For all of the examined cations, the equilibrium duration was discovered to be 60 min.

#### Effect of temperature

**Fig. 10:**
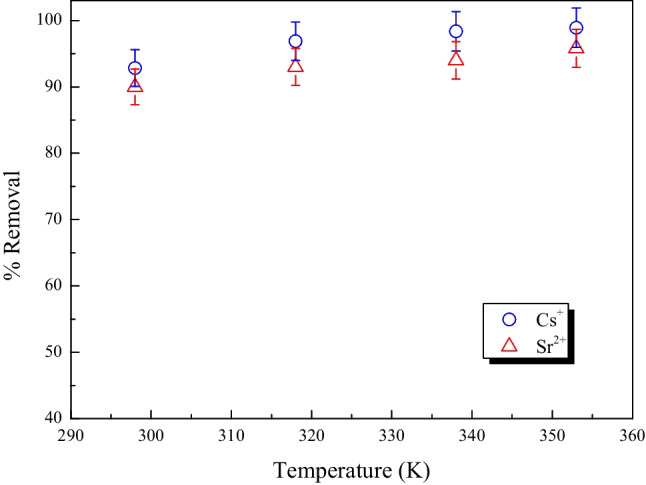
The effect of temperature on removing Cs^+^ and Sr.^2+^ by MoTiWPO_4_

A study of the effects of various temperatures was conducted at 298, 318, 338, and 353 K. The performance of the MoTiWPO_4_ across the investigated temperature range is shown in Fig. 10. It was evident that the percentage removal increased as the temperature is raised. This would suggest that Cs^+^ and Sr^2+^ions adsorbed onto MoTiWPO_4_ has an endothermic character. At an equilibrium duration of 60 min, a rise in temperature from 298 to 353 K causes an increase in the percent removal for Cs^+^ and Sr^2+^ indicating a temperature-dependent mechanism. This effect may be explained by the availability of additional adsorbent active sites, pore expansion, and/or activation of the adsorbent surface at higher temperatures. This might be owing to the increased mobility of Cs^+^ and Sr^2+^ ions from the bulk solution to the adsorbent surface, which increased penetration inside MoTiWPO_4_ (Mahrous et al. 2019; Mansy et al. 2023; Șenilă et al. 2023).

### Saturation capacity and thermal effect

The saturation capacity of MoTiWPO_4_ for Cs^+^ and Sr^2+^ is 113.47 and 109.11 mg/g, respectively. The thermal stability of MoTiWPO_4_ was studied as a function of saturation capacity for Cs^+^ ions, and this was achieved by heating the prepared sample at different drying temperature from 50 to 700 ± 1°C, after that, the capacity of MoTiWPO_4_ for Cs^+^ ions was determined by batch experiment studies; the data are recorded in Table 5. The data illustrates that the prepared MoTiWPO_4_ has good thermal stability as it retained about 97.77% of its saturation capacity for Cs^+^ ions by heating at 100 ± 1 °C, 92.44% by heating at 300 °C ± 1, 75.53% by heating at 500 ± 1 °C, and about 60.05% by heating at 700 ± 1 °C. The saturation capacity of MoTiWPO_4_ was found to decrease with increasing temperature from 500 to 700 ± 1 °C as observed in Table 5, and this is due to the loss of free and bonded water molecules which may be regarded as the exchangeable active sites (El-Naggar et al. 2014). This behavior is compatible with the obtained results by El-Naggar et al. (2010), El-Naggar et al. (2014), and Tourky et al. (2016).

**Table 5:** Effect of temperature on saturation capacity of MoTiWPO_4_ for Cs^+^ ions

Heating temperature (°C)	Cs^+^ sorption capacity (mg/g)	(%) capacity loss	%Retention
50	113.47	0	100
100	110.93	2.23	97.77
300	104.89	7.55	92.44
500	85.69	24.47	75.53
700	68.14	39.95	60.05

### Radiation stability

Using a Co-60 gamma-ray irradiator at a dosage rate of 10 kGy/h and absorbed doses of 50, 100, and 150 kGy, the impact of radiation on MoTiWPO_4_ was studied. The saturation capacity of the material for Cs^+^ before and after irradiation has been used to assess radiation stability. It was measured and its values are shown in Table 6. It was found that MoTiWPO_4_ has good radiation stability, losing only around 5.12, 6.4, and 10.61% of its capacity when exposed to radiation at doses of 50, 100, and 150 kGy, respectively (Abdel-Galil et al. 2018). As a result, MoTiWPO_4_ is thought to be an effective adsorbent for the removal of ^137^Cs and ^85^Sr radionuclides from radioactive waste solution.

**Table 6:** Effect of irradiation doses on the capacity of MoTiWPO_4_ for Cs^+^ at a dose rate of 10 kGy/h

Gamma-ray doses (kGy)	Capacity (mg/g)	Capacity loss (%)	Capacity retention (%)
0	113.47	0	100
50	108.34	5.12	94.88
100	107.06	6.4	93.59
150	102.85	10.61	89.39

### Distribution studies

**Fig. 11:**
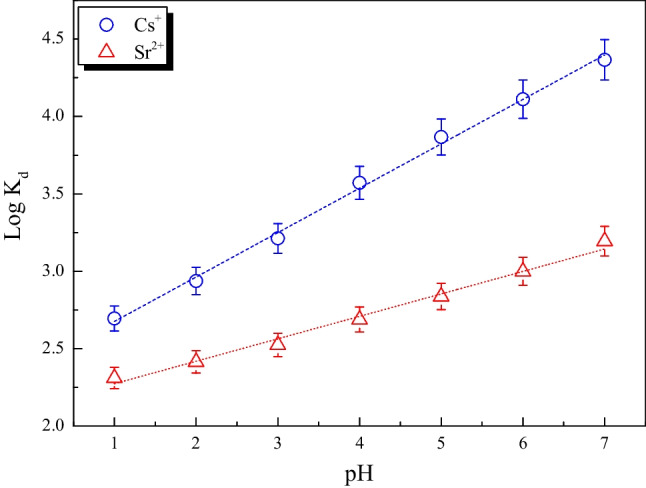
Log (*K*_*d*_) as a function of pH for Cs^+^ and Sr^2+^ on MoTiWPO_4_

The distribution coefficient (*K*_*d*_) of Cs^+^ and Sr^2+^ ions on MoTiWPO_4_ was calculated using batch equilibration as a function of pH. The values of the distribution coefficient (*K*_*d*_) were assessed using the following equation:

7 $${K}_{d}=\left[\left({C}_{i}-{C}_{f}\right)/{C}_{f}\right]\left(V/m\right)$$

where *C*_i_ and *C*_f_ are the concentrations of the ions in the solution before and after equilibration, respectively. Plotting the log *K*_*d*_ values vs. the pH of the solutions results in a straight line with a slope (*n*). The findings are shown in Fig. 11 and Table 7, which show how the pH affects the *K*_*d*_ values of the metal ions under study. As shown in Fig. 11 and Table 7, the *K*_*d*_ values of Cs^+^ and Sr^2+^ ions in the mono-component system increase with the increasing pH of the solution. At low pH, the concentration of hydrogen ions in the solution rises, potentially impeding the mobility of Cs^+^ and Sr^2+^ ions. In addition, a repulsive interaction was created between the positively charged surface of MoTiWPO_4_ and Cs^+^ and Sr^2+^ ions in the solution (Abdel-Galil et al. 2018). The sorption of Cs^+^ and Sr^2+^ ions became more favorable when pH increased due to the presence of an attractive force between the negatively charged surface and these positively charged ions (Abdel-Galil et al. 2018)**.** The best sorption occurs at pH 7, with removal percent and *K*_*d*_ values for Cs^+^ and Sr^2+^ ions of 99.57% and 23210 mg/L and 94% and 1566 mg/L, respectively. For the ions, Cs^+^ and Sr^2+^ the slopes of the linear correlations between log *K*_*d*_ and pH are 0.3 and 0.15 respectively. These slopes do not match the valences of the metal ions that have been sorbed, demonstrating the non-ideal ion exchange process.

**Table 7:** *K*_*d*_ values of Cs^+^ and Sr^2+^ on MoTiWPO_4_

Metal ions	pH of the solutions	*K*_*d*_, mL/g	Ionic radii, Å
Cs^+^	7	23,210.25	1.67
Sr^2+^		1566.67	1.18

### Thermodynamic parameters

**Fig. 12:**
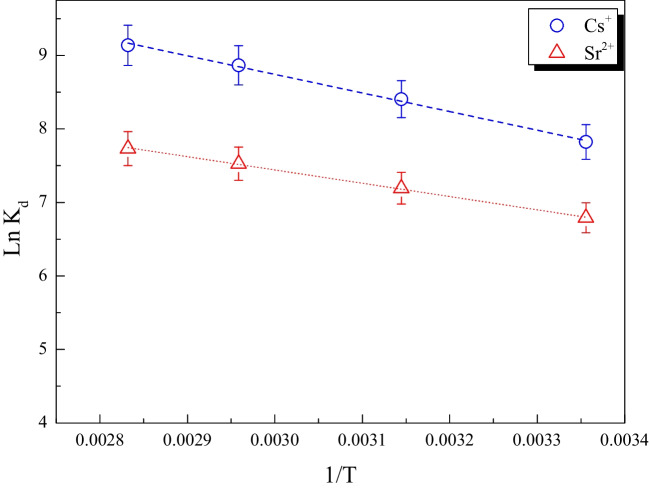
Ln (*K*_*d*_) as a function of 1/T for Cs^+^ and Sr^2+^ on MoTiWPO_4_

By examining the distribution coefficient *K*_*d*_ of Cs^+^ and Sr^2+^ ions sorbed on MoTiWPO_4_ at various temperatures (298, 318, 338, and 353 K), thermodynamic behavior was investigated. The following equations:

8 $$\Delta {G}^{o}=-RT\mathrm{ln}\left({K}_{d}\right)$$

9 $$\mathrm{ln}{K}_{d}=\frac{\Delta {\text{S}}^{o}}{R}-\frac{\Delta {H}^{o}}{RT}$$

10 $$\Delta {G}^{o}=\Delta {H}^{o}-T\Delta {\text{S}}^{o}$$

were used to compute several thermodynamic parameters, including the standard free energy change (*∆G°*, kJ mol^−1^), standard enthalpy change *(∆H°*, kJ mol^−1^), and standard entropy change (*∆S°*, J mol^−1^K^−1^) determined from the slope and intercept of the straight line produced by the plot of ln *K*_*d*_ as a function of 1/*T* (Fig. 12). Table 8 presents the thermodynamic outcomes. The positive value of Δ*H°* denotes the endothermic nature of the adsorption process, and the positive value of Δ*S°* indicates that the surface randomness increases during the adsorption process. The examined metal ions displayed negative free energy change (Δ*G*°) values, demonstrating the spontaneity of the adsorption process and the preference of these cations to adsorb on MoTiWPO_4_. Additionally, the fact that the negativity of *∆G°* rises as the temperature rises suggests that the adsorption process is endothermic and spontaneous (Lagergreen 1907; Olabemiwo et al. 2017)**.**

**Table 8:** Thermodynamic parameters for sorption of Cs^+^ and Sr^2+^ by MoTiWPO_4_

Ions	Temp. (K)	Δ*H*° (kJ mol^−1^)	Δ*G*° (kJ mol^−1^)	Δ*S*° (J mol^−1^ K^−1^)
Cs^+^	298	37.86	− 17.09	184.4
	318		− 21.25	
	338		− 24.44	
	353		− 26.70	
Sr^2+^	298	6.45	− 17.46	80.25
	318		− 19.02	
	338		− 20.67	
	353		− 22.69	

To compare the present work sorbent material performance with other materials, from the literature review, a comparison for the sorbent materials for different radionuclides is listed in Table 9.

**Table 9:** Comparison between sorbent materials for the removal of some radionuclides

Ref	Sorbent material	pH	Removal efficiency			
			^137^Cs	^134^Cs	^90^Sr	^85^Sr
Present work	MoTiWPO_4_	7.0	92.5	–-	–-	90.30
Abdel-Galil et al. (2019)	SnSiMo	5.0	96.5	–-	92.70	–-
Ali et al. (2020)	Dowex-HCR resin	10–11	–-	–-	–-	84.0
Attallah et al. (2016)	PAA/MA/SiO_2_/Al_2_O_3_	–-	–-	77.8	–-	–-

### Application

To test the ability of the prepared material (MoTiWPO_4_) in the efficient removal of the studied radionuclides from low-level liquid radioactive waste sample containing ^137^Cs and adding activated radionuclides such as ^60^Co, ^152^Eu, and ^85^Sr to simulate the competing ions, radiological experiments were conducted. Before carrying out the application, a radiological characterization of the radioactive waste sample was done using non-destructive gamma-ray spectrometry. The detector was coaxial-P-type HPGe (GEM-series, ORTEC, USA) with efficiency up to 30% and a resolution of 2.0 keV for 1173 and 1330 gamma energies. The detector has been calibrated for energy and efficiency to easily calculate the radioactivity of both cesium and strontium. The radioactivity level of the radioactive waste sample was determined by the following equation given by Hilal et al. (2022) and El Afifi et al. (2023) as

11 $$A\left(Bq/L\right)=\frac{\text{Net Counts-Background}}{t\times \varepsilon \times {I}_{\gamma }\times V}$$

In which *A* is the radioactivity level (Bq/L), *t* is the counting time, *ε* is the detector efficiency, *I*_*γ*_ is the probability of emission of each gamma ray, and *V* is the sample volume in (L). The radiological characterization of the sample reveals that each 10 mL from the sample contains 150 kBq from ^137^Cs, 31 kBq ^60^Co, 28 kBq ^152^Eu, and 74 kBq from ^85^Sr. Using a 15 mL-capacity polyethylene vial, mix 1.0 mL from the liquid waste with 9.0 mL distilled water and add 0.1 g from the MoTiWPO_4_ sorbent material. After that, radiometric counting was carried out to confirm the initial radioactivity level of the sample, then shaking them for 1 h.

**Fig. 13:**
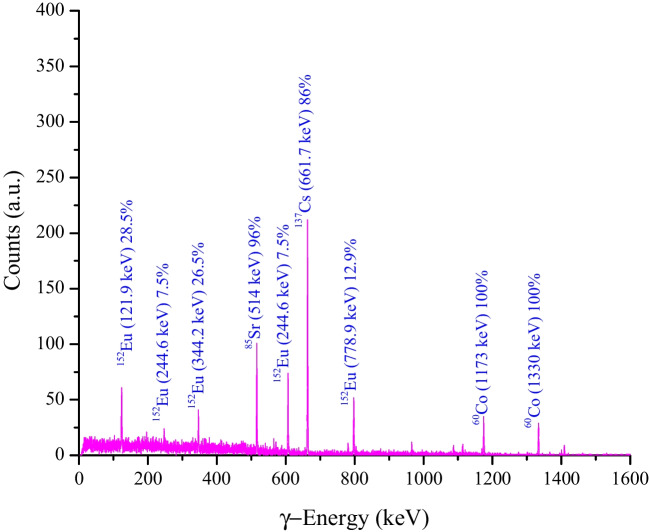
Gamma spectra of a radioactive waste characterization measured using HPGe detector giving the gamma energy line along with the probability of emission of each gamma energy

After settling by gravity, the decantation process was carried out to find the final radioactivity level of the sample. The result of counting the initial waste sample is plotted in Fig. 13, and the data obtained are listed in Table 10.

**Table 10:** Radioactivity levels of initial and final counting of the waste sample along with the decontamination factor and %eff

Radionuclides	Initial activity (Bq/mL)	Final activity (Bq/mL)	DF	% eff
^137^Cs	15,000 ± 122.47	1125 ± 33.5	13.3	92.5
^85^Sr	7,400 ± 86.02	717.8 ± 26.7	10.3	90.3
^60^Co	3,100 ± 55.57	558.0 ± 23.6	5.5	82.0
^152^Eu	2,800 ± 52.91	1190 ± 34.4	2.4	57.5

It is evident from Table 10 that MoTiWPO_4_ is proficient in removing both ^137^Cs and ^85^Sr from liquid radioactive waste samples by %eff. of 92.5 and 90.3, respectively, in the presence of competing ions from ^60^Co (divalent) and ^152^Eu (trivalent), confirming the batch experiment results for the removal of Cs^+^ and Sr^2+^ metal ions. In addition, the decontamination factor reaches 13.3 in the case of ^137^Cs and 10.3 for ^85^Sr.

## Conclusion

MoTiWPO_4_ showed promising results in removing Cs^+^ and Sr^2+^ metal ions from wastewater. It also showed that MoTiWPO_4_ has a suitable saturation capacity for Cs^+^ (113.47 mg/g) and Sr^2+^ (109.11 mg/g). The batch experiments found that the optimum adsorption equilibrium time was about 60 min at 298 K and pH = 7 for all studied metal ions. The experimental results show the high thermal stability of the sorbent material until 500 °C. Finally, MoTiWPO_4_ is a proficient sorbent material for removing both ^137^Cs and ^85^Sr from low-level-liquid radioactive waste samples by % eff. of 92.5 and 90.3, respectively, with high DF values.

## Data Availability

Yes.
